# Genome-wide association study for charcoal rot resistance in soybean harvested in Kazakhstan

**DOI:** 10.18699/VJGB-23-68

**Published:** 2023-10

**Authors:** A. Zatybekov, S. Abugalieva, S. Didorenko, A. Rsaliyev, A. Maulenbay, C. Fang, Y. Turuspekov

**Affiliations:** Institute of Plant Biology and Biotechnology, Almaty, Kazakhstan; Institute of Plant Biology and Biotechnology, Almaty, Kazakhstan; Kazakh Research Institute of Agriculture and Plant Growing, Almalybak, Almaty Region, Kazakhstan; Research Institute for Biological Safety Problems, Gvardeiskiy (Otar), Zhambyl Region, Kazakhstan; Research Institute for Biological Safety Problems, Gvardeiskiy (Otar), Zhambyl Region, Kazakhstan; School of Life Sciences, Guangzhou University, Guangzhou, China; Institute of Plant Biology and Biotechnology, Almaty, Kazakhstan

**Keywords:** soybean, charcoal rot, whole genome resequencing, GWAS, SNP, QTL, соя, пепельная гниль, полногеномное ресеквенирование, ПГАА, SNP, ЛКП

## Abstract

Charcoal rot (CR) caused by the fungal pathogen Macrophomina phaseolina is a devastating disease affecting soybean (Glycine max (L.) Merrill.) worldwide. Identifying the genetic factors associated with resistance to charcoal rot is crucial for developing disease-resistant soybean cultivars. In this research, we conducted a genome-wide association study (GWAS) using different models and genotypic data to unravel the genetic determinants underlying soybean resistance to сharcoal rot. The study relied on a panel of 252 soybean accessions, comprising commercial cultivars and breeding lines, to capture genetic variations associated with resistance. The phenotypic evaluation was performed under natural conditions during the 2021–2022 period. Disease severity and survival rates were recorded to quantify the resistance levels in the accessions. Genotypic data consisted of two sets: the results of genotyping using the Illumina iSelect 6K SNP (single-nucleotide polymorphism) array and the results of whole-genome resequencing. The GWAS was conducted using four different models (MLM, MLMM, FarmCPU, and BLINK) based on the GAPIT platform. As a result, SNP markers of 11 quantitative trait loci associated with CR resistance were identified. Candidate genes within the identified genomic regions were explored for their functional annotations and potential roles in plant defense responses. The findings from this study may further contribute to the development of molecular breeding strategies for enhancing CR resistance in soybean cultivars. Marker-assisted selection can be efficiently employed to accelerate the breeding process, enabling the development of cultivars with improved resistance to сharcoal rot. Ultimately, deploying resistant cultivars may significantly reduce yield losses and enhance the sustainability of soybean production, benefiting farmers and ensuring a stable supply of this valuable crop.

## Abstract (ru)

Пепельная гниль, вызываемая грибным патогеном Macrophomina phaseolina, представляет собой опасное заболевание, поражающее сою (Glycine max (L.) Merrill.) во всем мире. Выявление генетических факторов, связанных с устойчивостью к пепельной гнили, имеет важное значение для создания устойчивых к болезням сортов сои. Мы провели полногеномный анализ ассоциации (ПГАА) с использованием различных моделей и генотипических данных, чтобы найти генетические детерминанты, лежащие в основе устойчивости сои к пепельной гнили. В исследовании использовали коллекцию, состоящую из 252 образцов сои, включая коммерческие сорта и селекционные линии, для выявления генетических вариаций, связанных с устойчивостью. Фенотипическую оценку проводили в естественных условиях в период 2021–2022 гг. В работе регистрировали уровень заболевания и показатели выживаемости для количественной оценки уровней устойчивости образцов. Генотипические данные состояли из двух наборов: результаты генотипирования с применением технологии Illumina iSelect 6K SNP, и данные полногеномного ресеквенирования. Полногеномный анализ ассоциации был выполнен с помощью четырех различных моделей (MLM, MLMM, FarmCPU и BLINK) на платформе GAPIT. В результате были идентифицированы SNP-маркеры 11 локусов количественных признаков, ассоциированных с устойчивостью к пепельной гнили. Гены-кандидаты в пределах идентифицированных геномных областей были изучены на предмет их функциональной аннотации и потенциальной роли в защитных реакциях растений. Результаты этого исследования могут внести дополнительный вклад в разработку стратегий молекулярной селекции для повы- шения устойчивости сортов сои к пепельной гнили. Маркер-опосредованный отбор может быть эффективно применен для ускорения процесса селекции, что позволит создавать сорта с повышенной устойчивостью к пепельной гнили. Использование устойчивых сортов может значительно сократить потери урожая и повысить устойчивость производства сои, что принесет пользу фермерам и обеспечит стабильное производство этой ценной культуры

## Introduction

Soybean (Glycine max (L.) Merrill.) is one of the most important legumes in the world due to the high nutritional value and protein content of seeds (Pratap et al., 2016). According to the Agencies for Strategic Planning and Reforms of the Republic of Kazakhstan Bureau of National Statistics, soybean was grown on 127.7 thousand hectares in Kazakhstan in 2022 (https://new.stat.gov.kz/ru/industries/business-statistics/ stat-forrest-village-hunt-fish/publications/5099/). To further develop the soybean industry in Kazakhstan, the Government of Kazakhstan has announced a new program known as “Northern Soybean” to expand the soybean area to 1.5 million hectares (https://www.gov.kz/article/64601?lang=kk).

The important factor severely limiting soybean productivity is its susceptibility to harmful fungal diseases (Wrather et al., 2010; Bandara et al., 2020). Strategies for the management of soybean fungal diseases include cultural methods, seedapplied fungicides, and biological controls, but these have not been effective or widely adopted, and have provided limited control (Akem, 1996; Hartman et al., 2015). Therefore, genetic resistance may be the most feasible and sustainable method by which to manage fungal diseases (Lin et al., 2022). Breeding for resistance is difficult because most diseases are quantitatively inherited and controlled by multiple genes. However, modern genomic methodologies may help elucidate resistance mechanisms and identify resistant genotypes to improve breeding programs (St. Clair, 2010). For instance, breeding projects can be coupled with genome-wide association studies (GWASs), as this approach can efficiently identify candidate genes for disease screening.

Genome-wide association studies are becoming a routine approach in the search for marker-trait associations (Korte, Farlow, 2013) and can be efficiently applied to assess genetic variations of important agronomic traits, including disease resistance (Iquira et al., 2015). These studies use high-density single-nucleotide polymorphism (SNP) arrays and variable populations to enhance the mapping resolution, which drastically improves the identification of putative causal genes (Song et al., 2013; Zhang et al., 2015). Although there are possibilities in respect of GWASs for the prediction of false positive associations, applying variable statistical algorithms can be instrumental in controlling these. For instance, a mixed linear model (MLM) that uses population structure and kinship matrices will significantly regulate inflation (Kaler et al., 2020). However, this method may also remove true genes as background noise in certain studies of complex traits associated with the population structure. To overcome this obstacle, the Bayesian information and linkage-disequilibrium iteratively nested keyway (BLINK) employs a multiple-loci test method along with a fixed-effect model (FEM), Bayesian information criteria, and linkage disequilibrium information (Huang et al., 2019). Our previous GWAS of 182 soybean accessions for resistance to fungal diseases (Zatybekov et al., 2018) allowed the identification of 15 marker-trait associations (MTAs) for resistance to fusarium root rot (FUS, caused by Fusarium spp.), frogeye leaf spot (FLS, caused by Cercospora sojina), and brown spot (BS, caused by Septoria glycines).

In Kazakhstan, more than ten soybean fungal diseases have been identified (Mombekova et al., 2013; Didorenko et al., 2014; Zatybekov et al., 2018), and one category of these comprises root rot diseases (charcoal rot, phytophthora root rot, fusarium root rot, etc.). The expansion of these studies is an obvious necessity in order to examine the genetic background associated with the resistance to harmful pathogens in soybean. One of the local harmful root rot diseases is charcoal rot (CR) caused by Macrophomina phaseolina, a soil- and seed-borne polyphagous fungus (Paris et al., 2006).

Currently, only partial resistance has been recorded in soybean, while complete resistance to M. phaseolina has not been reported in any plant species (Paris et al., 2006; Pawlowski et al., 2015). In addition, it has been reported that no similar markers or genes have been found when comparing field and greenhouse studies, suggesting that CR resistance in soybean has a complex molecular mechanism. Therefore, the search for more valuable resistance sources from which to identify resistance genes should be continued. The main purpose of this study was to identify MTAs for charcoal rot resistance in a collection of 252 accessions from major soybean growing regions from around the world using the GWAS approach.

## Materials and methods

The soybean collection analyzed in this study consisted of 252 accessions, including 31 released cultivars and prospective breeding lines from Kazakhstan (Zatybekov et al., 2020). Accessions from 20 countries were represented in the collection and separated into five origin groups: Western and Eastern Europe, North America, East Asia, and Kazakhstan (Zatybekov et al., 2020).

The collection was assessed in the experimental plots of the Kazakh Research Institute of Agriculture and Plant Growing (south-eastern Kazakhstan) in the 2021–2022 period. Plants were grown in one-meter-long rows with a 30 cm distance between adjacent rows and 5 cm between plants within rows. The assessment of field resistance to CR was based on a generally accepted five-point scale: 1 – microsclerotia are not visible in the tissue (I-immune); 2 – very few microsclerotia are visible in the core, vascular tissue, or under the epidermis, and the vascular tissue has not changed color (R-resistant); 3 – vascular tissue is partially discolored, and microsclerotia partially cover the tissue (MR-moderately resistant); 4 – vascular tissue is discolored with numerous microsclerotia embedded in the tissue, and microsclerotia are also visible under the outer epidermis in stem and root sections (MS-moderately susceptible); and 5 – vascular tissue darkened due to the large amount of microsclerotia both inside and outside the tissues of the stem and root (S- susceptible) (Mengistu et al., 2007). The field experiments for CR resistance were conducted in triplicate and in randomized order. The results were analyzed using Statistical Package for the Social Sciences (SPSS 22.0.0.0) (https:// www.ibm.com/analytics/data-science/predictive-analytics/ spss-statistical-software) computer programs

The genotyping data consisted of two sets of SNP data. The first set (Set 1) was developed using the soybean 6K SNP Illumina iSelect array (Song et al., 2013) at Traitgenetics GmbH (Gatersleben, Germany). DNA samples were extracted and purified from single seeds of individual cultivars using commercial kits (Qiagen, CA, USA). The DNA concentration for each sample was adjusted to 50 ng/μl. SNP genotype analysis was carried out using Illumina Genome Studio software (GS V2011.1). The second set (Set 2) was developed at the Department of the School of Life Sciences, Guangzhou University, China, using whole-genome resequencing (WGRS) technology based on the Illumina HiSeq X Ten system (Lu et al., 2020). For each of the accessions in the panel of 252, at least five μg of DNA was used to construct a sequencing library with an Illumina TruSeq DNA Sample Prep Kit, according to the manufacturer’s instructions.

The SNP datasets were filtered using a 10 % cutoff for missing data, and markers with minor allele frequency ≥ 0.05 were considered for the genome-wide association studies. Numbers of hypothetical groups ranging from k = 1 to 10 were assessed using 50,000 burn-in iterations followed by 100,000 recorded Markov-chain iterations. The sampling variance of the population structure inference was estimated for each k using STRUCTURE software (Pritchard et al., 2000) with five independent runs. The delta K value (ΔK) was estimated using Structure Harvester (Evanno et al., 2005). The Q-matrix was developed based on the final k-values. Population genetic analysis was conducted using two sets of SNP data to construct a neighbor-joining tree with TASSEL software and further visualization using the iTOL online platform (https:// itol.embl.de/).

The GWAS for soybean resistance to CR in Southeast Kazakhstan was conducted using MLM (Yu et al., 2006), a multiple-locus mixed linear model (MLMM) (Segura et al., 2012), fixed and random model circulating probability unification (FarmCPU) (Liu et al., 2016), and BLINK models (Huang et al., 2019) using GAPIT V3 software (Wang, Zhang, 2021). The rMVP package (Yin et al., 2021) was used for the visualization with P ≤ 0.0002 thresholds. A QQ plot was applied to evaluate the distribution of observed p-values compared to the expected distribution under the null hypothesis of no association between genetic markers and the trait of interest (Ehret, 2010).

## Results

Disease resistance

**Fig. 1.**
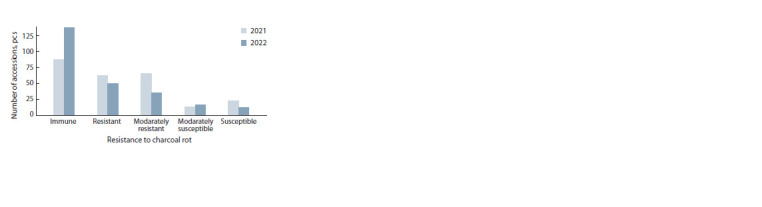
Resistance of the soybean collection to charcoal rot in the Southeast region of Kazakhstan.

Field trial results obtained at the experimental stations of the Southeast region suggested a clear difference in the development of charcoal rot. During the two-year period, the spread and effect of CR pathogens on plants were stronger in 2021 (Fig. 1, Supplementary Material 1)1. The results indicate that the group of susceptible accessions comprised 23 samples (9.1 %) in 2021 and 12 samples (4.8 %) in 2022 (see Fig. 1).

Supplementary Materials are available in the online version of the paper: https://vavilovj-icg.ru/download/pict-2023-27/appx21.pdf


Five genotypes were susceptible to resistance to CR during the two years of the study. Among them were three accessions from East Asia, i. e., cultivars Jin nong 62, Dong doe 027, and Mei feng 18 from China, and line 1034 from Korea. In addition, two accessions from East Europe (Osobliva from Ukraine and CH 147020-1 from Belarus) were susceptible to charcoal rot

Genetic variation in the soybean collection based on two sets of SNP markers

The final data after filtering consisted of 4495 (Set 1) and 44,385 (Set 2) polymorphic SNPs. Set 1 consisted of 77.98 % transitions and 22.02 % transverse variants, and Set 2 comprised 71.88 % transitions and 28.12 % transverse variants. The average length of chromosomes was 47.4 Mb for both sets, and the average number of SNPs per chromosome was 222.1 for Set 1 and 2219.3 for Set 2. The chromosome length ranged from 37.3 Mb in Gm16 to 62.1 Mb in Gm18 for Set 1, and from 34.7 Mb in Gm11 to 58 Mb in Gm18 for Set 2. The number of markers per chromosome varied from 185 in Gm11 to 295 in Gm13 for Set 1, and from 1438 in Gm11 to 3438 in Gm18 for Set 2. The average density of the SNP map was one marker every 213 Kb for Set 1 and every 22 Kb for Set 2.

**Fig. 2.**
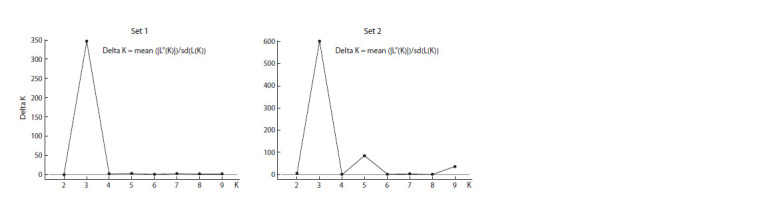
Determination of delta K (ΔK) using Structure Harvester software based on two genotypic datasets. Set 1 – data based on genotyping using Illumina iSelect array (4651 SNP); Set 2 – data based on resequencing technology (44,385 SNP).

The Structure Harvester results divided the studied collection into three clusters based on data from Set 1 and Set 2 (Fig. 2).

**Fig. 3.**
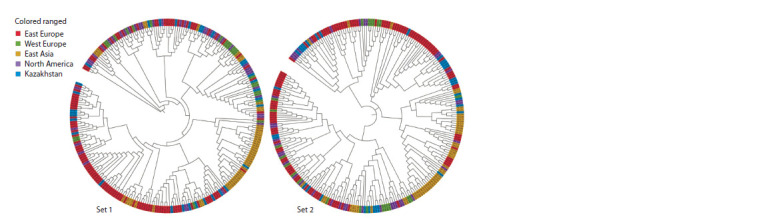
Neighbor-joining tree based on two sets of genotypic data. Set 1 – data based on genotyping using Illumina iSelect array (4651 SNP); Set 2 – data based on resequencing technology (44,385 SNP).

No clear separation of accessions depending on the origin was recorded, regardless of the set chosen. The largest group of accessions was distributed in the third cluster, which consisted of 165 genotypes in Set 1 and 168 samples in Set 2. Based on two sets of genotyping data, a phylogenetic tree was constructed using the neighbor-joining method (Fig. 3). Population analysis based on resequencing data showed a clear division into four populations (see Fig. 3, Set 2).

Association mapping

The GWAS of soybean resistance to CR was conducted using two sets of genotypic data (Set 1 and Set 2) and four models (MLM, MLMM, FarmCPU, and BLINK). The results of the GWAS using four models are shown in Supplementary Material 2. The comparative results of the associations suggest that the most informative results were obtained using the MLMM, FarmCPU, and BLINK models (Table 1).

**Table 1.**
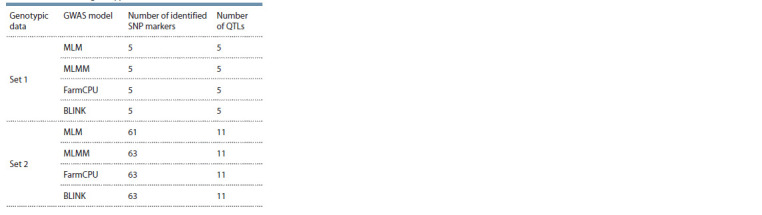
Comparison of identified numbers of SNPs and quantitative trait loci (QTLs) according to GWAS models based on two sets of genotypic data

The application of BLINK using Set 1 allowed the identification of five quantitative trait loci (QTLs) associated with CR resistance, while the usage of Set 2 expanded the detection to 11 QTLs that were significant at the threshold of P ≤ 0.002 (see Supplementary Material 3, Table 1). The QQ plot confirms the reliability of the associations (see Supplementary Material, 3, b).

The physical position of each critical SNP marker in the MTAs was overlaid with the positions of known QTLs (https:// soybase.org/search/qtllist_by_symbol.php) (Table 2). Particularly, the assessment of the 11 QTLs listed in Table 2 indicated that 8 of them were reported in published reports in respect of plant resistance studies.

**Table 2.**
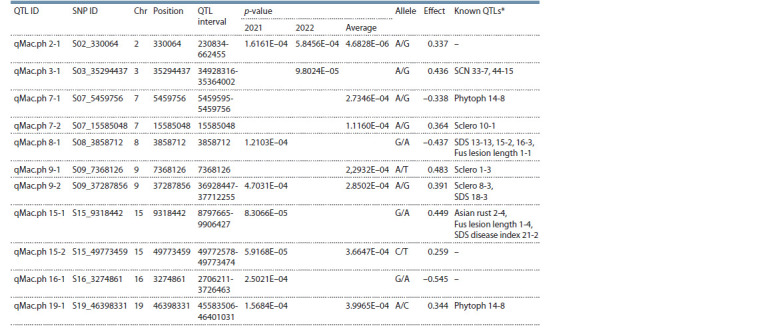
The list of identified QTLs using the BLINK model * Based on the QTL list on SoyBase (https://soybase.org/search/qtllist_by_symbol.php).

The largest numbers of QTLs associated with CR resistance using Set 2 were identified in chromosomes 7, 9, and 15 (Supplementary Material 4). Analysis of the genome physical locations of associated SNP markers revealed that all identified SNPs were part of the coding DNA sequence (CDS) (Table 3).

**Table 3.**
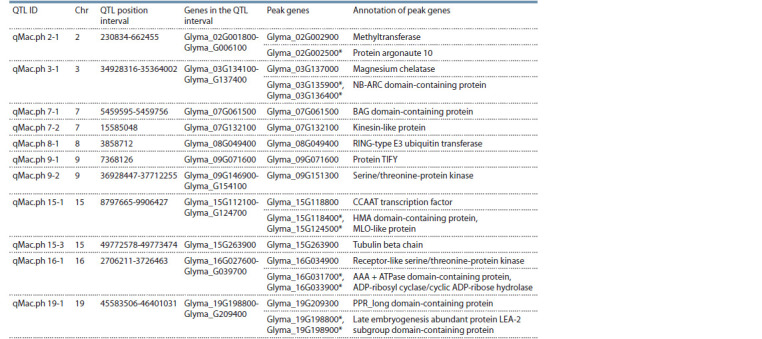
Physical positions of identified QTLs in the soybean genome * Genes associated with deseases resistance function

Each SNP in an intergenic position was considered for possible functional annotation based on the proximity of closely located genes.

## Discussion

The analysis of soybean CR development in Southeast Kazakhstan revealed a strong environmental influence on the distribution of pathogens and plant tolerance to the disease. Previous CR resistance studies in soybean were unsuccessful in identifying major genes that completely resist this pathogen (Coser et al., 2017), indicating that the resistance is quantitative and may rely on the efficiency of minor QTLs. Therefore, searching for resistant genotypes and applying powerful genomic tools are the obvious priorities for successful CR resistance breeding in soybean. The current work was conducted to identify QTLs associated with CR resistance in soybean using a genome-wide association studies.

The study was based on using two genotyping sets (Set 1 and Set 2) that drastically differed in the number of SNPs in the same soybean collection. Set 1 included 4651 SNPs and was extensively used in our previous GWAS projects (Zatybekov et al., 2020), while Set 2 included 44,385 SNPs, which is roughly ten times higher than the number in Set 1. As expected, the power of the GWAS using Set 2 (11 QTLs) was higher than that of Set 1 (five QTLs) (see Table 1) and relied on WRGS technology, which allows more in-depth searching and discovery of new genes associated with agronomic traits, as well as the study of evolutionary mutations in the genome. Most of the identified statistically significant QTLs were detected using the BLINK and FarmCPU models, which were applied among four different statistical approaches, including MLM and MLMM. In this study, BLINK and FarmCPU were the most successful approaches (see Supplementary Material 2), and additional estimations, including QQ plots, confirmed that these models generally result in fewer false positives and identify more true positives (Huang et al., 2019).

Assessment of the identified QTL locations showed that, in most cases, they are distant from each other, or detected on different chromosomes. Analysis of the SNPs in the identified MTAs revealed nine proteins associated with the immune response to pathogens. The qMac.ph 3-1 interval contains two genes (Glyma_03G0135900 and Glyma_03G0136400), both coding NB-ARC domain-containing protein, which is associated with resistance to fungal pathogens (Van Ooijen et al., 2008). The qMac.ph 15-1 interval contains gene Glyma_15G118400 coding HMA domain-containing protein, which is associated with regulation of the defense response to fungi.

In addition, the gene Glyma_15G124500, which codes MLO-like protein associated with powdery-mildew resistance (Shen et al., 2012), is also located in this interval. The qMac. ph 16-1 interval contains two genes, Glyma_16G031700 and Glyma_16G033900, associated with the defense response. The late embryogenesis abundant protein LEA-2 subgroup coding two genes, Glyma_19G198800 and Glyma_19G198900, was located in an interval of qMac.ph 19-1 and is associated with disease resistance. These examples of SNPs in MTAs, including presumably newly identified genetic factors that have not been matched to previously reported factors in the literature, require additional validation studies. Thus, the identified QTLs may facilitate the discovery of new genes for disease resistance and a better understanding of genotype × environment interaction patterns. The identified SNP markers (see Tables 2 and 3) for each of the detected QTLs of CR resistance can be efficiently used in marker-associated selection projects in soybean.

## Conclusion

Our GWAS of soybean resistance to CR has provided important insights into the genetic determinants underlying resistance to this devastating disease. By employing two sets of genotypic data with different SNP density levels and utilizing four GWAS models, we identified eleven QTLs that were statistically associated with CR resistance. The GWAS revealed the complexity of the genetic architecture underlying resistance to CR, indicating the involvement of multiple genes and molecular pathways. The identified genomic regions can serve as valuable targets for further functional validation and exploration of their specific roles in plant defense responses against Macrophomina phaseolina. Overall, the study represents a significant step in understanding the genetic basis of soybean resistance to charcoal rot. The knowledge gained from this research may further contribute to developing resilient soybean cultivars, ensuring a stable and sustainable supply of this essential crop while minimizing the economic and environmental impacts of charcoal rot.

## Conflict of interest

The authors declare no conflict of interest.
